# A randomized controlled trial examining the impact of diversified nursing interventions on the circadian rhythm of sedated critically ill patients

**DOI:** 10.3389/fmed.2025.1655844

**Published:** 2026-01-12

**Authors:** Mengfei Zhang, Aili Shi, Chen Xia, Liping Du, Shuangqin Zheng, Yong Nan, Zhen Cheng

**Affiliations:** 1 Intensive Care Unit, Emergency and Critical Care Center, Hangzhou Medical College, Zhejiang Provincial People’s Hospital (Affiliated People’s Hospital), Hangzhou, Zhejiang, China; 2 Department of Nursing, Emergency Center, Hangzhou Medical College, Tongde Hospital of Zhejiang Province, Zhejiang Provincial People’s Hospital (Affiliated People’s Hospital), Hangzhou, Zhejiang, China; 3 Department of Orthopedics, Center for Rehabilitation Medicine, Hangzhou Medical College, Zhejiang Provincial People’s Hospital (Affiliated People’s Hospital), Hangzhou, Zhejiang, China; 4 Department of Emergency Medicine, Emergency and Critical Care Center, Hangzhou Medical College, Zhejiang Provincial People’s Hospital (Affiliated People’s Hospital), Hangzhou, Zhejiang, China

**Keywords:** circadian rhythm, intensive care unit, melatonin, nursing, randomized controlled trial

## Abstract

**Background:**

Circadian rhythms are cyclical processes within an organism that develop over time for the organism to better adapt to its environment. Disruption of the circadian rhythm by various factors can have both acute and chronic adverse effects on the health of the organism. Sedated patients in the intensive care unit often have severe circadian rhythm disturbances, in which care-related exogenous factors play an important role.

**Methods:**

In this study, a randomized controlled trial design was adopted. Starting from the exogenous factors of nursing, the research objects that met the inclusion criteria were numbered. According to the inclusion order, they were odd numbered into the control group (receiving routine nursing care) and even numbered into the experimental group (receiving diversified nursing intervention). The two groups of research objects were divided into different sedation degree groups according to Richmond Agitation Sedation Scale (RASS) scores, which were divided into RASS 0–2 group, RASS -3–4 group and RASS -5 group, a total of six groups. The influence of diversified nursing intervention measures on patients’ circadian rhythm was evaluated by measuring the melatonin levels of the two groups.

**Results:**

We found that for patients with RASS scores of -3 to -4, diversified nursing interventions had a significant effect on melatonin concentrations in the 00:00–04:00 period (*P* < 0.05), indicating that diversified nursing interventions had a beneficial role in treating circadian rhythm disorders among sedated ICU patients.

**Conclusion:**

The circadian rhythm is crucial for critically ill patients and can be improved through diversified nursing interventions. This study provides relevant data for the future treatment of patients presenting with circadian rhythm disorders in the intensive care unit.

## 1 Introduction

Circadian rhythms are defined as human behavioral and physiological activities that involve repetitive 24 h cyclic rhythms and are integral to the normal survival of all living things on earth (1). The physiological activities of the human body, including brain arousal, sympathetic nerve tone, cardiovascular function, coagulation, immune system activity, metabolism, respiration, sleep cycles, and endocrine signaling, are subject to and play important roles in the cyclical variation of circadian rhythms. Disturbance of the circadian rhythm has an enormous effect on body systems (2, 3). The effects of circadian rhythms on human sleep and cognition are known to be the most common, and a retrospective study by Gao and Knauert (2) suggested that patients with circadian rhythm disorders tend to suffer from sleep disturbances, a process that exacerbates patients’ poor prognostic disease outcomes. In addition, sleep disruption caused by circadian rhythm disruption can also have adverse cognitive and emotional effects on patients. A study by Korompeli et al. (4) suggested that circadian rhythm disruption leads to disorientation, an inability to maintain attention, impaired short-term memory, and impaired visuospatial abilities in affected individuals. All of these processes have enormous impacts on a person’s normal physical life. With further research in recent years, scholars have also reported that circadian rhythm disruption can lead to serious organic diseases, as evidenced by cardiovascular function problems, such as increased susceptibility to ventricular arrhythmias (1), increased thrombosis, and an increased incidence of acute infarctions (5). Additionally, Li et al. (6) reported that patients with circadian rhythm disorders have a significantly higher mean arterial pressure at night, which in turn leads to greater mortality. Circadian rhythm disruption not only affects the cardiovascular system but also has a substantial effect on immune system homeostasis, and in patients with circadian rhythm disruption, there is an increase in inflammatory markers in the body and a decrease in acute-phase reactants, which ultimately leads to immunocompromise (2). Additionally, an article published by Poole and Kitchen (7) suggested that innate immune cells contain a molecular biological clock of rhythmic responses; from the size of the inflammatory response to the number of circulating immune cells, there is circadian variation throughout the day, which leads to circadian variations that have an impact on immune system-related disorders. Indeed, other systems, such as the digestive system and the endocrine system, are also regulated by circadian rhythms, and disruption of circadian rhythms can cause dysfunctions in related systems (2, 8). In summary, almost every system in the human body is affected by circadian rhythm disruption, which shows that a stable circadian rhythm is very important.

Numerous studies have demonstrated that intensive care unit patients often suffer from severe circadian rhythm disturbances (2, 9, 10). The problem of circadian rhythm disruption in intensive care unit patients is often caused by a combination of factors; ICUs differ from general wards in that patients are more severely ill, and doctors and nurses are often required to provide complex medical and nursing care to these individuals, which can lead to numerous external (environmental) and internal (medical) factors triggering the development of circadian rhythm disorders in patients (1). Specifically, prolonged confinement of critically ill patients in intensive care units (ICUs) and their exposure to externally imposed fixed environments (constant light, noise, frequent nursing activities, etc.) are highly likely to induce disruptions in circadian rhythms, which ultimately have a significant negative impact on these patients (11).

First, light plays an important role in circadian rhythm disruption in ICU patients, and the stability of circadian rhythms is significantly correlated with the dose, intensity (lux), and duration of light in the environment (2). Continuous light is generally required in the ICU setting for nursing care, which can severely affect the stabilization of patients’ circadian rhythms.

Second, noise decibels also have a strong impact on the circadian rhythms of ICU patients, with existing studies showing that noise levels in hospitals can be as high as 55–65 decibels or even as high as 80 decibels (4). In recent years, some studies have reported that the average level of noise in the ICU environment is 53–59 dB, and the peak level of noise is 67–86 dB because of factors such as the sound of staff conversations and activities, the sound of various instrument alarms, and the sound of nursing interventions and treatments (10). These noises affect the quality of sleep in critically ill patients, and the presence of such sounds is inevitably also a negative factor in the stabilization of the patient’s circadian rhythm, which in turn creates a vicious cycle.

In addition, the absence of rhythmicity in some operations of care in the intensive care unit, such as enteral nutritional support and patrolling modalities, can affect the circadian rhythm homeostasis of patients. A Yale University review of enteral nutrition support for critically ill patients mentioned that the most common form of enteral nutrition in ICUs at this stage involves starting patients on enteral nutrition pumps providing enteral nutrition, which is usually infused continuously at a constant rate for up to 24 h (12). The same approach to enteral nutrition was used in our central care unit and emergency intensive care unit, as described in the Yale University review, except for the use of an oral approach to autonomous enteral nutrition in awake patients who could feed on their own. Continuous enteral nutrition is contrary to the rhythmic nature of the body’s food intake, and the lack of rhythmic food intake has a strong effect on circadian rhythms as well. A study by Oyama et al. (13) suggested that irregular feeding leads to disruption of circadian rhythms and increased inflammatory responses in the body, which can be demonstrated by increased levels of interleukin (IL)-6 and tumor necrosis factor (TNF)-α and increased mortality. Marik (14) noted that in humans, continuous eating enhances insulin resistance, hyperglycemia, liver inflammation, and impaired bowel function.

Additionally, therapeutic maneuvers by nurses in routine ICUs are more common, and Knauert et al. (15) mentioned that nocturnal nursing activities lead to disruption of patients’ sleep, which in turn leads to disruption of their circadian rhythms.

Currently, circadian rhythms are assessed mainly by measuring the core body temperature and the levels of melatonin and cortisol (1, 9, 16). Melatonin levels have been shown to be the gold standard of measurement when studying circadian rhythm disorders (17, 18); therefore, this paper focuses on the use of melatonin levels as an indicators of circadian rhythm.

Although the potential negative effects of the above exogenous factors (light, noise, nutritional mode, nursing operation, etc.) on the circadian rhythm of ICU patients have been recognized, there are still some gaps in the current research. First of all, most studies focus on the intervention of a single factor, while the ICU environment is a complex system of multiple factors, and systematic and comprehensive nursing intervention research for multiple exogenous factors is not sufficient. Secondly, for a specific group of sedative patients in ICU, their self-regulation ability is weaker, and they are more vulnerable to exogenous factors. However, there is a relative lack of high-quality research on the impact of comprehensive nursing interventions (such as joint regulation of light, sound, nutrition and workflow) on the core indicators of circadian rhythm (such as melatonin) for this group.

Therefore, this study aims to fill this knowledge gap. We hypothesized that for sedated patients in ICU, the implementation of a diversified nursing intervention program integrating light management, noise control, intermittent enteral nutrition and optimizing the nursing workflow could effectively improve their circadian rhythm disorder. This study used a randomized controlled trial design, with serum melatonin level as the gold standard to measure the circadian rhythm, in order to systematically evaluate the clinical efficacy of this diversified intervention program on the circadian rhythm of patients with different degrees of sedation, in order to provide practical and feasible nursing practice reference based on evidence-based medicine for improving the prognosis of patients with sedation in ICU.

## 2 Materials and methods

### 2.1 Study design

This study is a single center, randomized controlled trial. The patients were randomly divided into control group and experimental group by 1:1 distribution ratio. The random sequence was numbered by independent statisticians, odd numbered into the control group, and even numbered into the experimental group. The two groups of subjects were managed in the same ward, clinical operation nurses and doctors have completed unified training on intervention measures and blood sample collection, but are not aware of the research purpose; Data analysts do not participate in the experimental process, but only collect and process data; In addition, the enrolled patients did not know the purpose of the study and informed their authorizers. Patients who met the inclusion criteria and were not subjected to nursing interventions from January 2023 to December 2023 were selected. The inclusion criteria for the patients were as follows: (1) ≥ 18 years of age and (2) sedation in our medical-surgical intensive care unit or emergency intensive care unit. Sedated patients were scored according to the Richmond Agitation Sedation Scale (RASS). The Ethics Committee of the Zhejiang Provincial People’s Hospital (Affiliated People’s Hospital), Hangzhou Medical College approved the research protocol of this study. Informed consent was obtained from patient’s families or their authorized persons for all treatments and indicators obtained.

Eligible patients were admitted to ICU for more than 48 h without diversified nursing intervention, and received routine nursing. The duration of the experimental intervention: 48 h after enrollment, lasting for 4 days. The exclusion criteria for patients were as follows: were younger than 18 years of age; were visually and hearing impaired; had severe sleep disorders; had a history of psychiatric disorders; had a preadmission occupation that required shift work; had a recent history of travel across two to three time zones; and had a history of alcohol and drug abuse. Patients who presented with severe craniocerebral injury, coma (not medically induced), or persistent status epilepticus at the time of admission; patients who received targeted temperature management; and patients who required prone ventilation to ensure adequate oxygenation were also excluded. The patients included in this study received different degrees of drug sedation. Meanwhile, in order to avoid the influence of sedative drugs on melatonin levels, patients with the same degree of sedation were included in the same group, According to the degree of sedation, they were divided into control group RASS-5, control group RASS-3—-4, control group RASS 0—-2, experimental group RASS-5, experimental group RASS-3–4, experimental group RASS 0–2. According to the guidelines, delirium score should be evaluated when the degree of sedation is ≥ 2 points. Confusion Assessment Method of Intensive Care Unit, CAM-ICU (CAM-ICU) score table was used to evaluate the delirium of patients and patients with delirium before and during the experiment were excluded.

Intervention measures: Patients were divided into a routine nursing group and an experimental group based on whether they receive diversified nursing interventions. The authorized persons of the research subjects participating in the experiment have all signed informed consent forms.

(Noun explanation) Diversified Nursing Interventions: Based on a literature review, diversified nursing interventions were designed and adopted in this study. Multivariate analysis refers to the analysis of corresponding nursing measures from the perspectives of light, noise, enteral nutrition, and workflow.

The intervention measures taken by the control group and the experimental group are shown in Table 1. Refer to Table 2 for the control of experimental intervention measures and personnel division involved.

**TABLE 1:** The intervention measures taken by the two experimental groups.

Diversified nursing interventions	Control (usual care) group	Experimental group
Illumination	White woven lights predominated during the day; Floor-to-ceiling window blinds closed and opened at times (irregularly and not for much time); White woven lights were used for most of the first half of the night, with lights out for some responsible groups in the second half of the night when the workload was low or there were no new admissions.	During the day, blinds were opened to increase the intensity and duration of light, and natural light was used whenever possible, provided that it was safe and protected patient privacy; Lights in the patient area were turned off after 9 pm to reduce light exposure; the brightness of the bedside computer monitoring screen was adjusted, and the patient wore an eye mask.
Noise	Routine care noise largely exceeded 50 dB during the day and 40 dB at night.	The volume of each instrument was adjusted at night (provided it was safe to do so) to minimize the noise generated by personnel activities; the patient wore earplugs.
Enteral nutrition modalities	Continuous nasogastric or nasoenteric tube feeding, depending on the patient’s total daily amount of enteral nutrition, the patient’s basic nighttime uninterrupted, with basic interruptions ranging from 0 to 5 h.	Intermittent daytime nasal feeding or oral enteral nutrition in accordance with normal eating habits, morning, midday and evening, with additional meals as needed at night for patients if needed (fasting at night if possible).
Medical nurse patient interaction	Complicated night shift handover process with frequent therapeutic maneuvers.	Therapeutic operations were completed during the day; patients were awakened daily as their condition permitted; daytime activities were increased; relaxing music was played in the ward, and family visits were implemented; the night shift handover process was optimized to reduce nighttime treatments.

**TABLE 2:** Detailed description of control and experimental interventions.

Component	Control group (routine care)	Experimental group (diversified nursing intervention)	Key collaborators and roles
Light management	- Primary use of white fluorescent lighting during daytime. - Irregular and limited opening of window blinds. - White lights often used during the first half of the night; lights might be turned off in the second half only if workload was low.	- Daytime: Blinds opened to maximize natural light exposure (safe & privacy permitting). - Nighttime (after 21:00): Overhead lights in patient area turned off. Bedside monitor brightness minimized. - Nighttime: Patients provided with eye masks.	Nurses: Primary executors. Doctors: Support the policy change.
Noise control	- Routine care noise, often exceeding 50 dB during day and 40 dB at night. No specific noise reduction protocols.	- Active Reduction: Nighttime volume of non-critical alarms reduced (safety permitting). Staff reminded to minimize noise. - Passive Protection: Patients provided with earplugs.	Nurses: Lead execution and reminders. Respiratory Therapists: Adjust ventilator alarms. All Staff: Compliance.
Enteral nutrition	- Continuous feeding via nasogastric/nasoenteric tube over 20–24 h, with minimal (0–5 h) interruption at night.	- Intermittent feeding administered during daytime hours (e.g., morning, noon, evening) to mimic normal eating patterns. - Nighttime fasting encouraged if clinically possible.	Doctors: Prescribe the change in feeding mode. Dietitians: Calculate the intermittent feeding regimen. Nurses: Administer and monitor tolerance.
Workflow and care activities	- Complex night shift handovers. - Frequent therapeutic maneuvers and patient interactions distributed throughout day and night.	- Daytime: Non-urgent procedures completed. Patients awakened daily if condition allowed. Daytime activities (e.g., passive/active exercises, family visits) were encouraged. - Nighttime (20:00–08:00): Care activities were clustered to maximize uninterrupted sleep periods. Night shift handover process was optimized to be quicker and quieter.	Nurses: Coordinate and schedule care. Doctors: Assess readiness for daily awakening and activity. Physical Therapists: May be involved in activity planning. Families: Participate in daytime visits.

### 2.2 Data analysis

Patients who met the inclusion criteria for the experiment and had been admitted for 48 h were divided into two groups on the basis of odd or even numbers (jointly completed by the research leader and data collector). The primary outcome was the 24-h circadian rhythm profile of serum melatonin concentration. Secondary outcomes included: (1) environmental light intensity; (2) environmental noise levels; (3) frequency of healthcare provider-patient interactions; (4) adherence to the assigned enteral nutrition protocol.

(1)    Hourly data on vital signs were collected from the study subjects in both groups from 48 h after admission to the 6th day after admission.
(2)    Sedation scores were recorded for each study subject at Q4h via the RASS Sedation Rating Scale, which was assessed daily at 9 am-1 pm-5 pm-9 pm-1 am-5 am in both our central care unit and the emergency care unit.
(3)    For melatonin measurement: Blood samples (3 mL) were drawn daily via an arterial line at 02:00, 04:00, 06:00, 08:00, 10:00, 12:00, 14:00, 16:00, 18:00, 20:00, 22:00, and 24:00. Samples were immediately centrifuged, and the plasma was stored at -80°C until analysis. Plasma melatonin concentrations were determined using a commercially available enzyme-linked immunosorbent assay (ELISA) kit according to the manufacturer’s instructions. All samples were analyzed in duplicate.
(4)    Measurements were made every 2 h in the middle of the patient partition in the intensive care unit using a Deli light detector, and the average value was taken as the light intensity measurement data.
(5)    Volume decibels were monitored for 24 h using a Deloitte noise decibel detector in the center of the intensive care unit, and data were recorded every 2 h.
(6)    The daily enteral nutrition duration in control patients was monitored.
(7)    The number of health care patient interactions per day at night and the average interval between them were monitored in both study groups.

Among the 110 patients included in the present study, 78 patients completed 4 days of data monitoring: 40 patients in the control group and 38 patients in the experimental group. Considering patient disease differences, individual factors influenced the irregular timing of the peak melatonin levels in many patients. We selected the highest value within the maximum average continuous series of the two sets of study data, excluding individual discrete data. In this study, the data were processed using SPSS 26.0, and graphs were created via GraphPad Prism. Quantitative information that conformed to a normal distribution is expressed herein as the mean ± standard deviation, and independent sample *t* tests were used to compare between-group differences in noise, light, and melatonin levels between the intervention and control groups. Paired sample *t*-tests were used to compare within-group differences. Qualitative information is expressed as the frequency (percentage); the chi-square test was used to compare the differences between the groups, and the difference was considered statistically significant at *P* < 0.05.

#### 2.2.1 Sample size calculation

Based on the effect quantity reported in the previous pre experiment or literature, the sample size was calculated by G * power software. It was concluded that at least 34 patients in each group were required, and the total sample size was 78 cases, which met the requirements.

#### 2.2.2 Data missing and processing methods

Data missing: among the 110 patients initially included, 32 patients dropped out due to rapid changes in disease condition, death or incomplete data collection, and the final completion rate was 70.9%.

Processing method: for a few scattered and randomly missing physiological parameter data points, we used the last observation value carry forward method to fill. For key outcome indicators (such as melatonin) that are measured multiple times as planned, we conducted a complete case study, that is, only patients with complete data at a specific time point were analyzed.

#### 2.2.3 Expansion of statistical methods and management of confounding factors

Beyond *t*-test and chi square test: we clearly pointed out that for the core outcome—repeated measurement of melatonin data, we used a more advanced “repeated measurement analysis of variance” or “linear mixed effect model.” These methods can consider time effect, inter group effect and their interaction at the same time, and are more suitable for analyzing longitudinal data.

Management of potential confounding factors: in order to control the impact of potential confounding factors such as age, gender and underlying diseases on outcomes, we conducted multivariate analysis of variance. We included these baseline variables as covariates in the model to evaluate the independent effect of the intervention after adjusting for these factors.

## 3 Results

1.    Table 3 Clinical characteristics of patients who completed the data monitoring are summarized below:
  The patients studied in this experiment were all ICU patients who needed sedation. In the experiment, we tried to choose the same sedative drug as much as possible based on the patient’s condition to reduce errors caused by different sedatives; In addition, statistics were conducted on whether mechanical ventilation was used. Although there were patients in both experimental groups who did not receive mechanical ventilation, the differences between the groups were within the standard error after statistical analysis. For the 78 patients who completed the data collection, there was no significant difference in age between the control group and the experimental group; 59.0% of these patients were male, and 41.0% were female. All patients were sedated medically, and 92.4% were mechanically ventilated; however, there were still more exposure variables, and patients on similar sedative medications were selected whenever possible. Propofol and midazolam were used in 42.3% of the patients, and propofol and dexmedetomidine were used in some of the patients, as shown in Table 3.

**TABLE 3:** Clinical characteristics of patients who completed data monitoring.

Variable		Total	Control group	Intervention group	*t*/χ ^2^	*P*
Age		70.44 ± 14.23	70.10 ± 15.01	70.79 ± 13.56	−0.212	0.832
Gender	Male	46(59.0)	23(57.5)	23(60.5)	0.074	0.786
	Female	32(41.0)	17(42.5)	15(39.5)		
RASS	0–2	18(23.1)	10(25.0)	8(21.1)	0.302	0.86
	3–4	51(65.4)	26(65.0)	25(65.8)		
	–5	9(11.5)	4(10.0)	5(13.2)		
Sedative drug grouping	Propofol	14(18.0)	9(22.5)	5(13.2)	3.019	0.697
	Midazolam	4(5.1)	3(7.5)	1(2.6)		
	Dexmedetomidine	7(9.0)	3(7.5)	4(10.5)		
	Propofol + Midazolam	42(53.9)	19(47.5)	23(60.5)		
	Propofol + Dexmedetomidine	8(10.3)	4(10.0)	4(10.5)		
	propofol + Midazolam + Dexmedetomidine	3(3.9)	2(5.0)	1(2.6)		
Reason for sedation	Severe pneumonia	11	5	6		
	Acute exacerbation of chronic obstructive pulmonary disease	14	5	9		
	Acute myocardial infarction	4	3	1		
	Acute heart failure	7	3	4		
	Aortic dissection	1	0	1		
	Sepsis	25	14	11		
	Multiple injuries (no head injury)	9	5	4		
	Severe pancreatitis	2	1	1		
	Postoperative (non-neurosurgical)	5	2	3		
Mechanical ventilation	No	5(6.4)	3(7.5)	2(5.3)	0	> 0.999
	Yes	73(93.6)	37(92.5)	36(94.7)		
Data loss reason	The change of illness did not meet the inclusion criteria	19	11	8		
	Transfer to another department	9	3	6		
	Discharge	2	2	0		
	death	2	1	1		

RASS, Richmond Agitation Sedation Scale.

**FIGURE 1:**
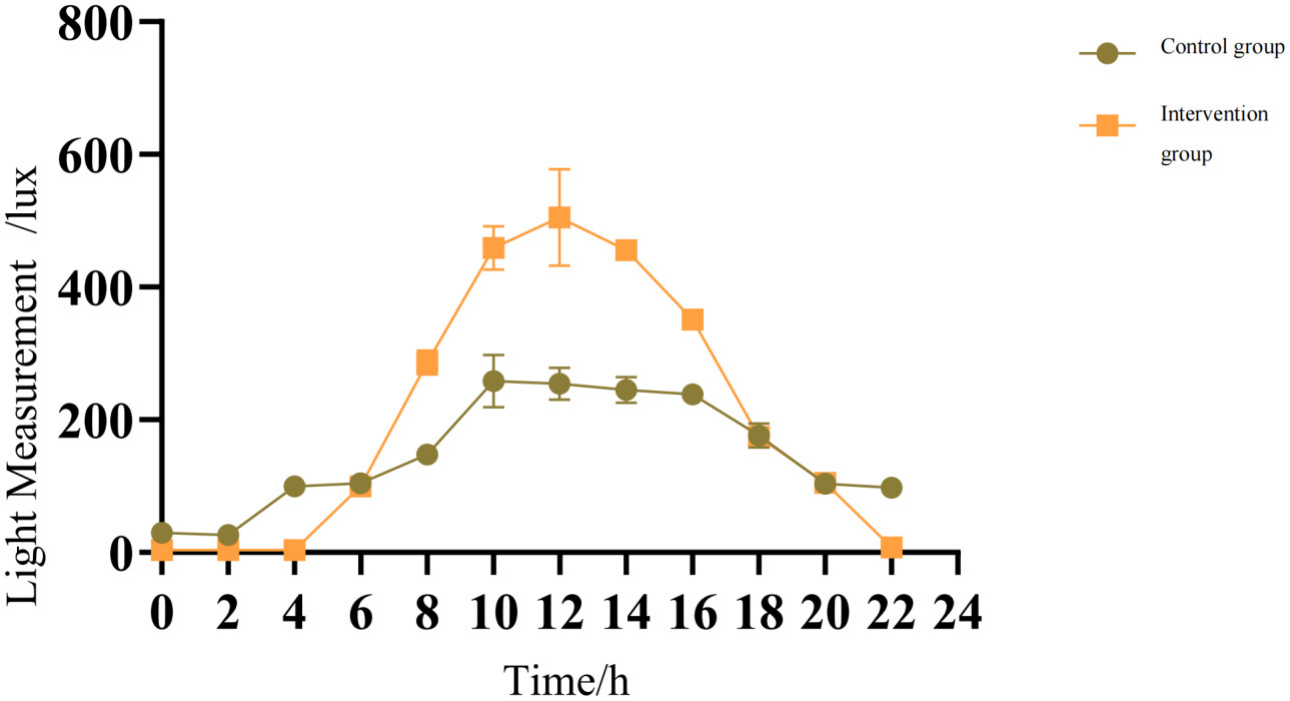
Light intensity monitoring data. Intensity of light in the intensive care unit measured in the two study groups.

2.    Considering that the light intensity taken at different locations is not the same, we chose the patient subregion right in the middle of the location of the measurement and took the average of the partition to derive the difference between the light levels of the two groups. As shown in Table 4 and Figure 1, the median light exposure in the environment where the critically ill patients were located showed a daily variation pattern (*p* < 0.001), the median daytime light intensity in the control group and *p* < 0.001 in the experimental group, and the control of light during the experiment was considered effective. The intensity of light at night was significantly greater in the control group than in the experimental group, which correlates with, among other things, the workflow of routine care in the control group, as also presented in the following table. Additionally, the light intensity changed significantly after the experimental group opened the blinds to allow natural light to enter.

**TABLE 4:** Light intensity monitoring data.

Time (24-h system)	Control group/Lux	Intervention group/Lux	*t*	*P*
time_0	29.75 ± 3.50	3.75 ± 0.96	14.331	**<0.001**
time_2	26.50 ± 6.86	3.75 ± 0.96	6.573	**0.001**
time_4	99.75 ± 1.71	3.75 ± 0.96	98.065	**<0.001**
time_6	104.00 ± 2.94	99.50 ± 1.29	2.800	**0.031**
time_8	147.75 ± 11.81	286.00 ± 18.35	–12.670	**<0.001**
time_10	258.00 ± 39.64	459.00 ± 32.65	–7.828	**<0.001**
time_12	254.00 ± 24.18	505.25 ± 72.88	–6.544	**0.001**
time_14	245.00 ± 19.15	455.50 ± 8.50	–20.093	**<0.001**
time_16	238.25 ± 4.99	350.75 ± 14.34	–14.820	**<0.001**
time_18	176.50 ± 18.16	174.50 ± 7.77	0.203	0.846
time_20	103.75 ± 3.30	104.75 ± 4.27	–0.370	0.724
time_22	97.75 ± 6.13	7.75 ± 1.71	28.284	**<0.001**

Intensity of light in the intensive care unit measured in the two study groups.

**FIGURE 2:**
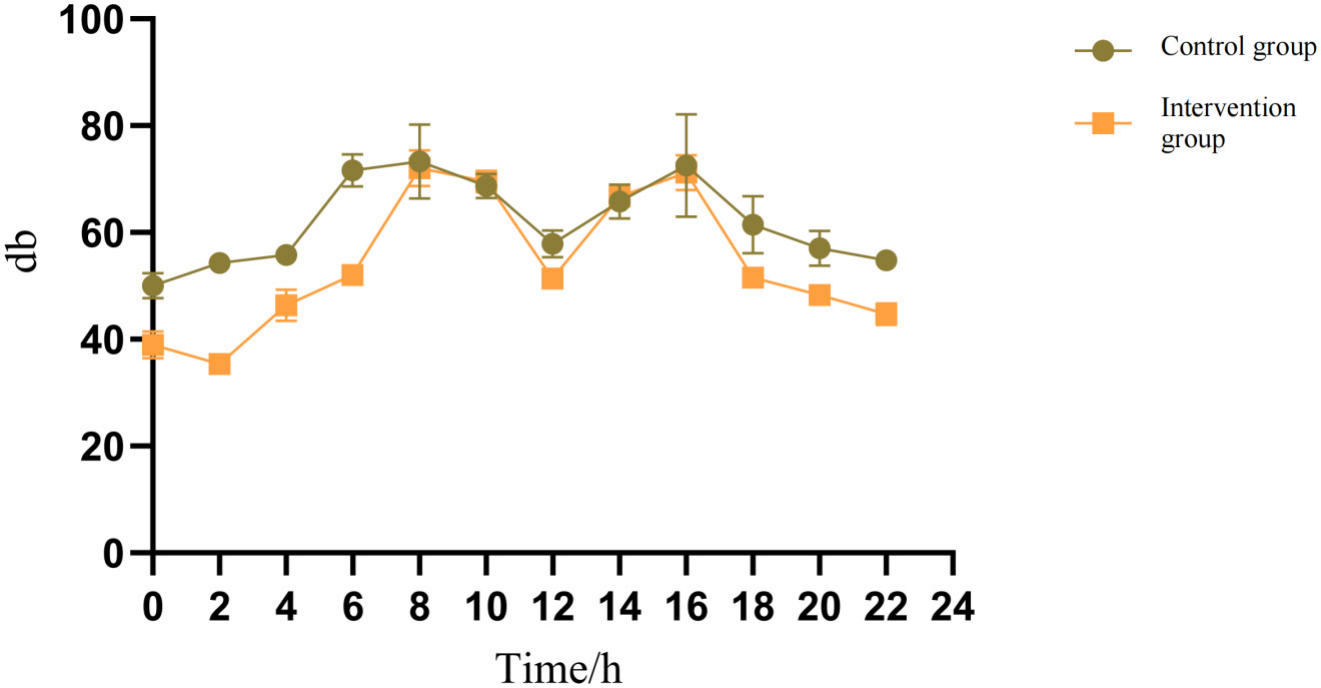
Noise monitoring data. Noise monitoring data directly in the middle of the delineated area of the intensive care unit where the two groups of study subjects were located.

3.    We selected the center of the monitoring room area location to assess the environmental factors affecting the control of noise, and the measured noise data are shown in Table 5 and Figure 2. The noise levels of the control and experimental groups were *p* < 0.001, and the control of noise during the experiment was also considered effective.

**TABLE 5:** Noise monitoring data.

Time (24-h system)	Control group/db	Intervention group/db	*t*	*P*
time_0	50.05 ± 2.34	38.98 ± 2.54	6.403	0.001
time_2	54.33 ± 1.85	35.38 ± 1.85	14.479	< 0.001
time_4	55.80 ± 1.52	46.40 ± 2.97	5.641	0.001
time_6	71.60 ± 3.01	52.03 ± 1.75	11.257	< 0.001
time_8	73.25 ± 6.92	72.08 ± 3.36	0.306	0.77
time_10	68.68 ± 2.25	69.57 ± 1.99	–0.599	0.571
time_12	57.85 ± 2.53	51.35 ± 1.30	4.568	0.004
time_14	65.75 ± 3.18	66.68 ± 1.71	–0.512	0.627
time_16	72.53 ± 9.59	71.20 ± 3.22	0.262	0.802
time_18	61.47 ± 5.34	51.58 ± 1.17	3.624	0.011
time_20	57.00 ± 3.25	48.25 ± 1.69	4.773	0.003
time_22	54.80 ± 1.85	44.70 ± 2.01	7.399	< 0.001

Noise monitoring data directly in the middle of the delineated area of the intensive care unit where the two groups of study subjects were located.

4.    In addition, the number of health care interventions was also determined, and there was a significant improvement in the number of interventions in the control and experimental groups after the improvement in the daily workflow, as shown in Table 6.

**TABLE 6:** Comparison of enteral nutrition modalities and the number of interactions.

	Control group	Intervention group	*t*	*P*
**Average number of interactions**				
Day	31.48 ± 6.97	31.08 ± 6.48	0.26	0.796
Night	21.53 ± 5.05	14.82 ± 2.20	7.53	<0.001
**Intermittent enteral nutrition**				
No	40(100.0)	0(0.0)	78.000	<0.001
Yes	0(0.0)	38(100.0)		

Comparison of the average number of interactions during the day (8 a.m.–8 p.m.) and the average number of interactions during the night (8 p.m.–8 a.m.) with a distinction between enteral nutrition modalities.

5.    All patients with severe sedation were categorized into control Group 1 (RASS 0–2), control Group 2 (RASS 3–4), control Group 3 (RASS-5), experimental Group 1 (RASS 0–2), experimental Group 2 (RASS-3–4), or experimental Group 3 (RASS-5) according to the RASS score. The data confirmed the presence of abnormal melatonin levels in sedated patients in the intensive care unit 48 h after admission to the unit and the presence of abnormal circadian rhythms in the patients, mainly in moderately and severely sedated patients, with a lesser impact on mildly sedated patients, especially those who were assessed as lucid by the assessment of their state of mind. The RASS-2 score of patients in the experimental group was higher than that of patients in the control group at 0:00 a.m.-4:00 a.m. in the melatonin segment, which demonstrated the effectiveness of this intervention in the experimental group. In addition to the measurements taken on the first day and the fourth day, the patient characteristics in the separate statistics revealed that there was no significant disruption of the circadian rhythm in mildly sedated patients, especially in lucid patients. In all three groups of patients with different degrees of sedation, it was also confirmed that during the day, the body’s melatonin levels were low and essentially stable and that the melatonin levels were at their highest peak from 2:00 to 4:00 a.m. The RASS-5 score experiment revealed circadian rhythm disturbances in both groups and no significant fluctuations in melatonin levels, which were not improved by the intervention. In contrast, there was a difference in the RASS-3–4 score; both groups had circadian rhythm disturbances in the first 24 h after 48 h of admission to the department, but with the experimental intervention, there was a significant fluctuation in the experimental group from 0:00 to 4:00 (*P* < 0.05) and a significant difference between the 4th and 1st days of the comparative measurements (*P* < 0.05). These findings indicated that diversified nursing interventions have an impact on circadian rhythms in patients with mild to moderate sedation, and that these impacts can be improved.

**FIGURE 3:**
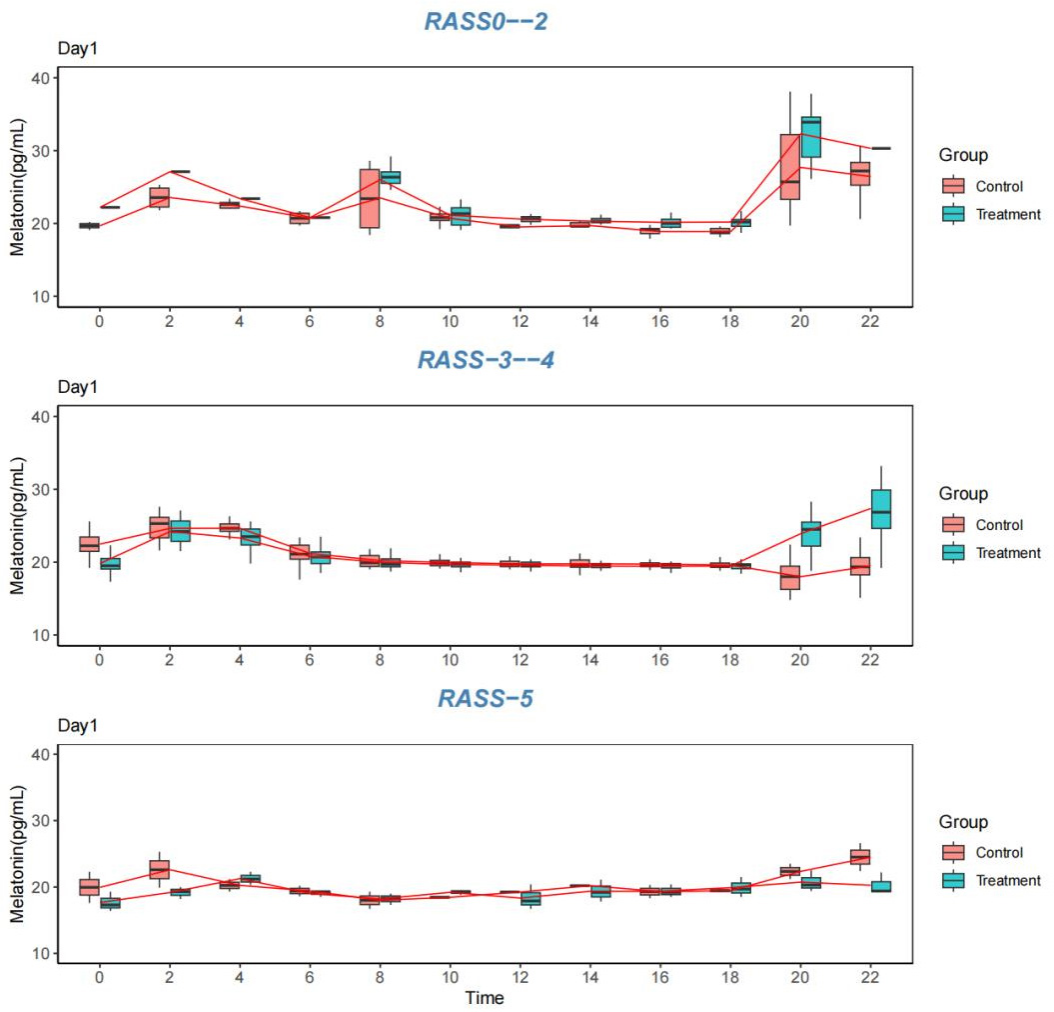
Melatonin levels. Comparison of melatonin levels between the two groups on the first day, grouped according to the RASS score. RASS, Richmond Agitation-Sedation Scale.

**FIGURE 4:**
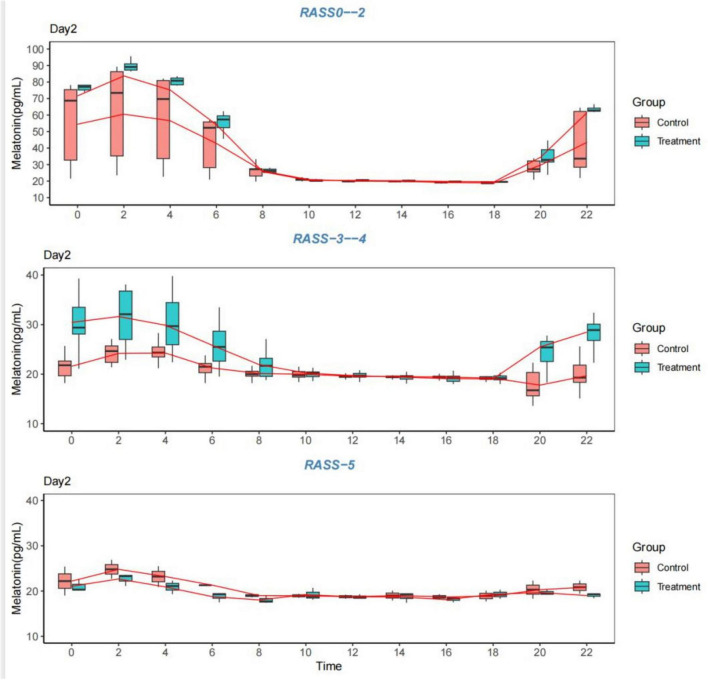
Melatonin levels. Comparison of melatonin levels between the two groups on the second day, grouped according to the RASS score. RASS, Richmond Agitation-Sedation Scale.

**FIGURE 5:**
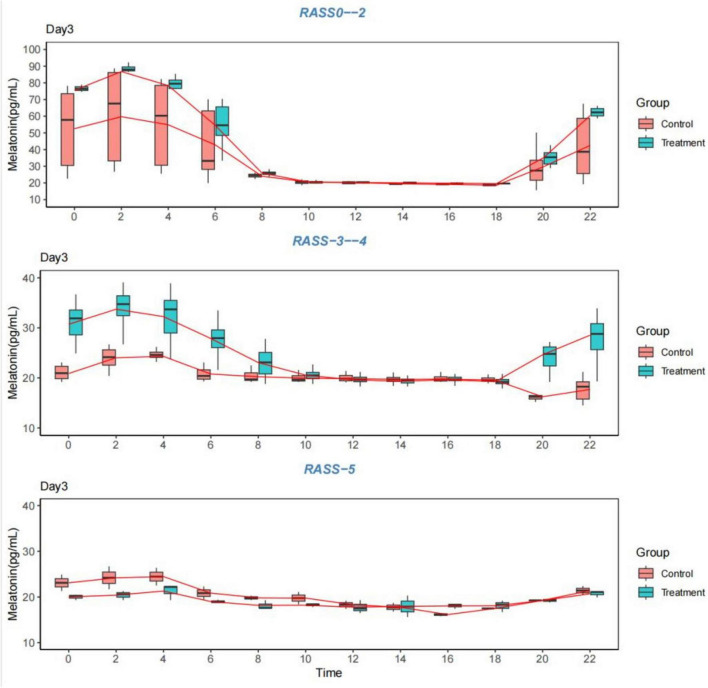
Melatonin levels. Comparison of melatonin levels between the two groups on the third day, grouped according to the RASS score. RASS, Richmond Agitation-Sedation Scale.

**FIGURE 6:**
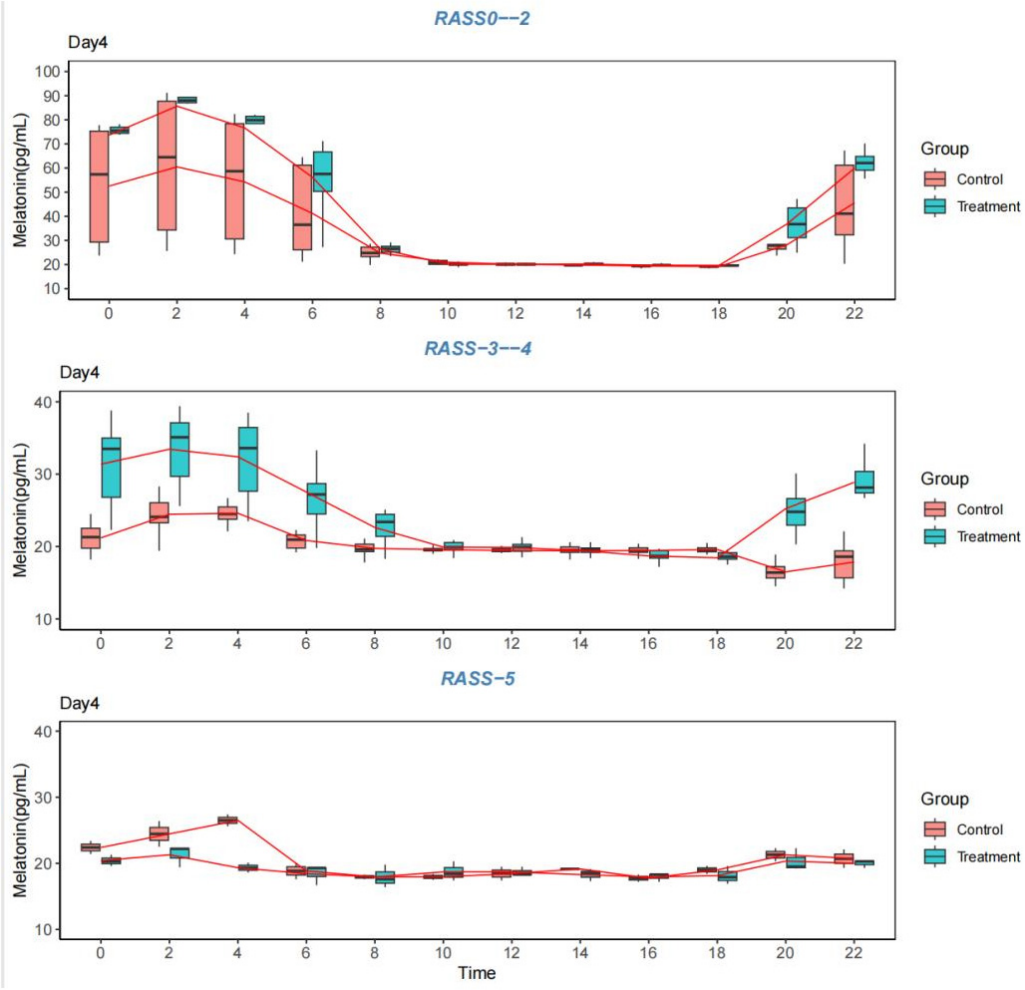
Melatonin levels. Comparison of melatonin levels between the two groups on the fourth day, grouped according to the RASS score. RASS, Richmond Agitation-Sedation Scale.

The data for 1–4 days from the start of the measurement were graphed as follows: Table 7; Figures 3–6.

**TABLE 7:** Melatonin levels.

RASS 0–2/RASS-3–4/RASS-5					
Time (24-h system)	Group	Day 1	Day 2	Day 3	Day 4
time_0	Control group	49.18 ± 28.14	54.43 ± 23.63*	52.54 ± 23.71	52.56 ± 23.06
	Intervention group	67.00 ± 18.38	71.59 ± 15.06*	75.54 ± 3.91**	73.68 ± 6.57**
time_2	Control group	58.26 ± 33.27	60.52 ± 27.16	59.72 ± 27.49	60.49 ± 27.43
	Intervention group	80.20 ± 21.54	83.70 ± 18.34	86.76 ± 6.04**	85.66 ± 9.31**
time_4	Control group	53.59 ± 29.87	56.57 ± 24.81	54.86 ± 24.39	54.24 ± 24.20
	Intervention group	73.11 ± 20.21	75.16 ± 16.31	78.36 ± 6.24**	76.63 ± 10.33**
time_6	Control group	42.16 ± 21.41	42.78 ± 14.81	42.84 ± 19.58	41.18 ± 17.57
	Intervention group	57.23 ± 15.31	54.13 ± 15.35	54.55 ± 13.38	56.13 ± 14.41**
time_8	Control group	23.50 ± 4.29	26.11 ± 3.96	24.02 ± 1.98	24.73 ± 2.77
	Intervention group	26.01 ± 2.60	25.71 ± 2.44	25.85 ± 1.39**	26.44 ± 1.87
time_10	Control group	20.69 ± 1.25	20.89 ± 0.95	20.43 ± 1.10	20.98 ± 0.90
	Intervention group	21.13 ± 1.47	20.39 ± 0.62	20.50 ± 0.72	20.14 ± 0.73
time_12	Control group	19.51 ± 0.63	20.00 ± 0.54*	20.16 ± 0.66*	20.10 ± 0.56
	Intervention group	20.60 ± 1.05**	20.44 ± 0.58*	20.28 ± 0.45	20.10 ± 0.47
time_14	Control group	19.70 ± 0.63	19.82 ± 0.29	19.46 ± 0.35	19.81 ± 0.43
	Intervention group	20.31 ± 0.95	20.16 ± 0.48	20.04 ± 0.50**	20.21 ± 0.55
time_16	Control group	18.88 ± 0.75	19.12 ± 0.42	19.09 ± 0.67	19.34 ± 0.57
	Intervention group	20.15 ± 0.81**	19.80 ± 0.28**	19.80 ± 0.39**	19.74 ± 0.49
time_18	Control group	18.88 ± 0.53	18.89 ± 0.39	18.61 ± 0.96	19.18 ± 0.54
	Intervention group	20.19 ± 0.94**	19.63 ± 0.38**	19.58 ± 0.48***	19.65 ± 0.60
time_20	Control group	27.69 ± 6.14	29.73 ± 7.89	29.70 ± 11.51	28.17 ± 5.80
	Intervention group	37.16 ± 7.88	34.53 ± 6.79	35.09 ± 4.96	36.80 ± 7.89**
time_22	Control group	45.22 ± 19.21	43.50 ± 18.44	42.54 ± 18.69	45.59 ± 17.77
	Intervention group	59.83 ± 12.21	61.46 ± 7.25**	60.41 ± 7.41**	59.96 ± 9.60
time_0	Control group	22.48 ± 1.72	21.60 ± 2.23	20.89 ± 1.78*	21.20 ± 1.91
	Intervention group	19.81 ± 1.43**	32.87 ± 8.91***	36.09 ± 10.72***	36.52 ± 11.60***
time_2	Control group	24.64 ± 2.25	24.20 ± 1.88	24.03 ± 1.81	24.46 ± 2.11
	Intervention group	24.20 ± 1.66	37.08 ± 11.26***	40.72 ± 11.56***	40.23 ± 12.41***
time_4	Control group	24.65 ± 1.81	24.26 ± 1.85	24.31 ± 1.57	24.60 ± 1.30
	Intervention group	23.31 ± 1.55**	34.54 ± 10.70***	37.94 ± 11.26***	37.63 ± 11.83***
time_6	Control group	21.19 ± 1.46	21.25 ± 1.46	20.78 ± 1.29	20.83 ± 0.97
	Intervention group	20.79 ± 1.24	26.92 ± 6.34***	28.54 ± 5.32***	29.47 ± 7.84***
time_8	Control group	20.22 ± 0.94	20.14 ± 1.03	20.23 ± 1.00	19.75 ± 0.79
	Intervention group	19.98 ± 0.97	21.94 ± 2.48***	23.07 ± 2.81***	22.63 ± 2.25***
time_10	Control group	19.94 ± 0.57	19.98 ± 0.81	19.96 ± 0.75	19.56 ± 0.63
	Intervention group	19.67 ± 0.75	20.23 ± 1.32	20.55 ± 1.03***	19.92 ± 1.10
time_12	Control group	19.76 ± 0.52	19.56 ± 0.79	19.84 ± 0.88	19.45 ± 0.68
	Intervention group	19.58 ± 0.53	19.72 ± 0.90	19.62 ± 0.75	19.88 ± 1.07
time_14	Control group	19.77 ± 0.71	19.54 ± 0.77	19.71 ± 0.62	19.40 ± 0.87
	Intervention group	19.46 ± 0.48	19.42 ± 0.80	19.33 ± 0.73	19.46 ± 0.77
time_16	Control group	19.73 ± 0.53	19.40 ± 0.40	19.77 ± 0.79	19.46 ± 0.58
	Intervention group	19.43 ± 0.52	19.09 ± 0.73	19.63 ± 0.84	18.71 ± 0.77***
time_18	Control group	19.59 ± 0.50	19.19 ± 0.75	19.56 ± 0.82	19.59 ± 0.43
	Intervention group	19.46 ± 0.56	19.05 ± 1.13	19.25 ± 0.82	18.45 ± 0.96**
time_20	Control group	17.99 ± 2.24	17.79 ± 2.73	16.24 ± 0.93*	16.50 ± 1.26*
	Intervention group	23.91 ± 3.63**	25.48 ± 5.88**	26.76 ± 7.56**	26.27 ± 6.36**
time_22	Control group	19.50 ± 2.24	19.69 ± 2.71	17.71 ± 2.02*	17.86 ± 2.18*
	Intervention group	28.37 ± 6.23**	31.97 ± 9.64***	31.68 ± 8.78**	32.37 ± 9.30***
time_0	Control group	19.95 ± 3.32	22.20 ± 4.53	23.10 ± 2.55*	22.40 ± 1.41
	Intervention group	17.67 ± 1.48	21.07 ± 1.42*	20.00 ± 0.62*	20.40 ± 0.85*
time_2	Control group	22.60 ± 3.82	24.80 ± 2.97	24.20 ± 3.54	24.45 ± 2.76
	Intervention group	19.17 ± 0.91	22.67 ± 1.37*	20.40 ± 1.01*	21.30 ± 1.65*
time_4	Control group	20.25 ± 1.34	23.20 ± 3.25	24.45 ± 2.76	26.50 ± 1.27
	Intervention group	21.27 ± 1.00	20.90 ± 1.51*	21.30 ± 1.73*	19.33 ± 0.75***
time_6	Control group	19.40 ± 1.13	21.30 ± 0.00	20.85 ± 2.05*	18.85 ± 1.77
	Intervention group	19.03 ± 0.46	18.77 ± 1.10*	19.00 ± 0.36*	18.43 ± 1.50*
time_8	Control group	18.00 ± 1.84	19.00 ± 0.57	19.80 ± 0.71*	17.95 ± 0.49*
	Intervention group	18.20 ± 0.85	18.03 ± 0.85*	18.17 ± 0.98*	17.93 ± 1.72*
time_10	Control group	18.45 ± 0.21	18.95 ± 0.64	19.75 ± 1.91*	17.95 ± 0.64*
	Intervention group	19.20 ± 0.36	19.20 ± 1.35	18.20 ± 0.35	18.70 ± 1.47*
time_12	Control group	19.25 ± 0.07	18.75 ± 0.64	18.30 ± 1.27*	18.45 ± 1.48*
	Intervention group	18.33 ± 1.89	18.73 ± 0.49	17.77 ± 1.42	18.70 ± 0.69
time_14	Control group	20.20 ± 0.14	19.00 ± 1.56	17.75 ± 1.34*	19.15 ± 0.07*
	Intervention group	19.37 ± 1.66	18.70 ± 1.13	17.93 ± 2.35*	18.30 ± 0.92
time_16	Control group	19.30 ± 1.41	18.70 ± 0.85	16.15 ± 0.49*	17.75 ± 0.78*
	Intervention group	19.37 ± 0.96	18.10 ± 0.52	18.03 ± 0.55**	17.93 ± 0.64*
time_18	Control group	19.45 ± 0.35	18.90 ± 1.70	17.50 ± 0.00*	19.00 ± 0.85*
	Intervention group	19.90 ± 1.51	19.27 ± 1.00	18.07 ± 1.27*	18.13 ± 1.37*
time_20	Control group	22.35 ± 1.63	20.30 ± 2.83	19.25 ± 0.07*	21.30 ± 1.41*
	Intervention group	20.73 ± 1.59	19.67 ± 0.64	19.23 ± 0.40	20.33 ± 1.71
time_22	Control group	24.50 ± 2.97	20.85 ± 2.05	21.40 ± 1.41*	20.70 ± 1.98*
	Intervention group	20.27 ± 1.68	19.00 ± 0.52*	20.70 ± 0.69	20.00 ± 0.61*

Melatonin levels in the two groups of study subjects according to the RASS Score. RASS, Richmond Agitation-Sedation Scale;

**p < 0.05 compared with the control group at the same time,

*p < 0.05 compared with Day 1 at the same time,

***p < 0.05 compared with the control group and Day 1 at the same time.

6.    In addition, we also conclude that, Light intensity data (Table 4): use independent sample *t*-test to compare the light intensity of the control group and the experimental group at different time points. The results showed that at night time points (such as time_22, i.e., 22:00), the light intensity of the experimental group was significantly lower than that of the control group (for example, time_22: control group 97.75 ± 6.13 lux vs. experimental group 7.75 ± 1.71 lux, *t* = 28.284, *p* < 0.001). This shows that the workflow intervention (lights out at night) effectively reduces the light intensity. Workflow data (Table 6): the average number of interactions at night in the experimental group was significantly lower than that in the control group (21.53 ± 5.05 times in the control group vs. 14.82 ± 2.20 times in the experimental group, *t* = 7.53, *p* < 0.001), which reflected that workflow optimization reduced night activities. Indirect correlation: Although the correlation coefficient between light intensity and work flow was not directly calculated, through the comparison between groups, the light intensity at night and the number of interactions at night in the experimental group decreased significantly after the implementation of workflow intervention and light intervention at the same time. This supports the operational correlation between light intensity changes and workflow interventions. That is, the adjustment of workflow (such as reducing night operation) is the direct reason for the reduction of light intensity.

With the present study, we confirmed that the original ICU model of light, noise, continuous enteral nutrition and frequent nocturnal provider–patient interactions had an impact on the circadian rhythms of sedated patients. Melatonin is the gold standard for measuring circadian rhythms; by regulating the above four major factors through diversified nursing interventions, the patients’ melatonin levels were changed. Therefore, we believe that diversified nursing interventions can ameliorate circadian rhythm disorders in sedated patients in the intensive care unit. In addition, the regularity of melatonin amplitude was evident in sedated awake patients, and the effects of the intensive care unit environment on these patients were relatively weak. Finally, in the context of combining other clinical characteristics, circadian rhythm disorders were present in sedated patients in the intensive care unit with different diseases, and no significant differences in their disorders were observed.

## 4 Discussion

The patients included in the present study were sedated in the intensive care unit and graded on a sedation scale to eliminate interference from the level of sedation. The diseases of patients in the intensive care unit are often complex and variable, and in addition to the influence of exogenous factors on circadian rhythms, endogenous factors, such as patient disease factors, pharmacologic factors, and mechanical ventilation patterns and parameters, likewise have an impact on these rhythms. Therefore, an experiment was conducted to minimize exposure to such variables and to analyze the clinical efficacy of diversified nursing interventions for sedated ICU patients from a macroscopic point of view, and the above-described conclusions were drawn.

In this study, we selected melatonin data from patients 48 h after admission and showed that the majority of critically ill patients receiving sedative medication 48 h after admission had circadian rhythm disturbances, which is consistent with the findings of Oxlund et al. (19). However, it has also been noted that, in severely neurologically impaired critically ill patients, there is a rapid disruption of melatonin immediately after admission, and with the severity of neurological disease, there is a delay and attenuation of melatonin; moreover, the changes in awake patients are almost not significantly different from those observed before admission (17). This finding is in line with the present study, where awake patients with a RASS score of 0–2 exhibited circadian rhythm homeostasis.

In this study, patients with sedation in the intensive care unit were mainly included, and their sedation scores were graded to eliminate the interference of sedation degree on the study. The diseases of patients in intensive care unit are often complex and changeable. In addition to the influence of exogenous factors on the circadian rhythm, endogenous factors also affect it, such as patient disease factors, drug factors, mechanical ventilation mode and parameters, etc. Therefore, when conducting the experiment, we should try to reduce the exposure of variables, and analyze the clinical efficacy of diversified nursing intervention on sedation patients in ICU from a macro perspective. However, the positive conclusions of this study must be interpreted with caution because of several important limitations.

First of all, although we grouped the sedation level by RASS score and selected patients using similar sedatives as much as possible, the main limitation of this study is that we failed to control the potential confounding factors by multivariate statistical models (such as multiple regression analysis). The circadian rhythm of critically ill patients is affected by a complex network, including but not limited to the severity of the disease (such as APACHE II or SOFA score), specific diagnosis (such as sepsis, acute respiratory distress syndrome), age, complications, organ failure status, the occurrence of delirium, and the types and doses of vasoactive drugs and antibiotics used. Although this study was partially controlled by exclusion criteria at the experimental design stage, in the statistical analysis, the potential effects of these factors on melatonin levels and final results could not be excluded by the inter group comparison (*t* test and chi square test). Therefore, although the inter group differences we observed are related to the intervention measures in time, they cannot be completely attributed to the intervention itself.

Secondly, there are potential sources of bias and inaccuracy in this study. Due to the characteristics of intervention measures (such as adjusting the light, wearing eye mask and ear plugs), although the nursing staff did not know the purpose of the study, they could not completely implement the blind method to the nursing staff and researchers, which may introduce implementation bias in data recording (such as noise, light measurement) and the implementation intensity of nursing measures. In addition, although we use the objective biomarker melatonin, its secretion is also affected by many of the above confounding factors, and its measured value may reflect the comprehensive effect of these factors, not just the effect of intervention measures. The sample size is relatively small, and there are patients falling out in the data collection (110 were included, 78 completed), which may also lead to selection bias and affect the statistical efficacy.

Despite these limitations, the findings of this study are consistent with a large number of existing literature reports, providing supporting evidence for the importance of exogenous nursing factors on circadian rhythm.

Most studies have shown that the rhythmicity of light is crucial for circadian rhythm stabilization. Gao and Knauert (2) and Fan et al. (20) argued that the circadian cycle and light/dark cycle of lighting are highly important; subsequently, researchers and scholars have suggested that the use of natural light as much as possible during the day in wards and eye masks worn at night can significantly improve patients’ circadian rhythms. Additionally, Khoddam et al. (21) noted that the use of eye masks was effective in controlling the circadian sleep rhythm in patients. Several studies have indicated that even exposure to low light levels (5–10 lux) while sleeping with one’s eyes closed at night can elicit a circadian rhythm response (10, 22). ICU patients are exposed to light for long periods, and the findings of the present study confirmed the effect of light on their circadian rhythm. The use of eye masks to minimize nocturnal light exposure in this trial is thought to further reduce light interference and demonstrates the effectiveness of this care measure. In addition to the effectiveness of the experimental treatment, there are still topics that need to be considered, such as the data collected in this study from the emergency intensive care unit. At the 1st floor location, the presence of green plants outside the window, especially on cloudy and rainy days with daytime lighting, have a certain impact.

Previous studies related to noise and circadian rhythms have suggested that wearing earplugs at night helps patients stabilize their circadian rhythms. Hu et al. (23) concluded that combining earplugs and eye masks with relaxing background music could help promote sleep circadian rhythms in adult patients in the CSICU. The present study confirmed the effect of noise on patients’ circadian rhythms and that the use of earplugs at night achieved better results, especially in moderately and heavily sedated mechanically ventilated patients who were in a passive position and had better compliance. In addition, some of the mildly sedated patients in this study reported wearing earplugs that still allowed ambient noise to be heard, caused discomfort while wearing them and dropped out after the patients fell asleep at night. In addition to the wearing of earplugs, the root cause of the problem was also addressed in this study by reducing environmental noise, such as reducing the sound of various instrument alarms under the premise of safety, which is thought to have a positive effect on patient sleep and circadian rhythms.

With respect to enteral nutrition and improving workflow, reducing the frequency of health care patient interactions, especially at night, is reported by most current studies to be beneficial for regulating circadian rhythm disorders in patients, although some studies on enteral nutrition in critically ill patients suggest that intermittent feeding leads to impaired gastric emptying, diarrhea, aspiration, and difficulty in controlling blood glucose (12). These conditions exist in some patients, but intermittent enteral nutrition is more closely associated with the body’s circadian rhythm mechanism (24). There were such patients in the present study who may have had complications due to the intermittent enteral nutrition approach, but the latest guidelines (25–27) suggest that intermittent feeding is preferable for critically ill patients whose conditions allow it. Intermittent feeding was used in this study and is considered to have a positive effect on circadian rhythm disruption. The complexity and variability of the condition of critically ill patients; the type of disease, such as diabetes; the severity of the disease; the use of vasoactive drugs, antibiotics, etc.; and intestinal infections may affect the outcome of enteral nutrition. In this study, there were also complications in patients who received continuous enteral nutrition or interrupted enteral nutrition, and these phenomena may have affected the results of the study and led to doubts about whether the findings are applicable to the majority of patients; therefore, additional studies are needed. In 2013, Davidson et al. (28) suggested that early activity helps normalize circadian rhythms in critically ill patients. The use of intensive care at night reduces sound, allows patients to rest longer, and contributes to the adjustment of their circadian rhythm (4, 28, 29). In this study, by optimizing the nursing process, consciously reducing the frequency of nurse–patient interactions at night, and appropriately increasing the daytime activities of mildly sedated patients, we concluded that this method is more in line with the laws of circadian rhythmic activities in the human body and has a positive effect on the stability of circadian rhythms.

However, circadian rhythms are also influenced by many other factors, including endogenous factors such as mechanical ventilation, and medication also negatively affects patients’ circadian rhythms (30). For example, the choice of different ventilator modes and parameters, the metabolic kinetics of the drugs and their duration of use, and the type of drugs, such as vasopressors, positive inotropes, and β-agonists, tend to increase melatonin levels, whereas β-blockers may inhibit the production of melatonin and therefore affect the patient’s circadian rhythms (31). There is also some information about the effects of sedative medications. A study by Jobanputra et al. (9) suggested that medications used for sedation, such as benzodiazepines or opioids, can alter the sleep–wake pattern in critically ill patients, thus affecting circadian rhythms. Patients in this study, especially those with mild sedation, were selected as much as possible without the use of catecholamine vasoactive drugs, and propofol and midazolam were used in deeply sedated patients to minimize variable exposure.

In the future, regulating circadian rhythm homeostasis may become an advantageous therapeutic measure for patients in intensive care units, and the use of diversified nursing interventions is essential for achieving individualized care for critically ill patients; however, a great deal of research and practice is needed in anticipation of the emergence of a model more conducive to circadian rhythm homeostasis in critically ill patients.

## 5 Limitations and future directions

There are several important limitations in this study, and we hope to continue to broaden and improve in the future research:

The data statistical model used in this study is relatively simple. Although patients are treated as a whole for nursing intervention, endogenous factors such as disease type, severity, age, complications, delirium, drug use and so on may affect melatonin levels;

The sample size of this study is relatively small, and it belongs to a single center study, hoping to expand the research scale in future experiments;

Although this study blinded the participating researchers and patients, the nursing intervention measures could not be completely blinded in the implementation process, and might also have a certain bias on the data.

In the future, adjusting the stability of the circadian rhythm may become a favorable treatment measure for patients in the intensive care unit. The key to realize the individualized nursing of critically ill patients is to adopt diversified nursing intervention measures; However, it needs a lot of research and practice, even combined with the big data model, and more diversified data statistical analysis methods are used to monitor and adjust different diseases, and generate the regulation mode assisted by artificial intelligence, in order to produce a model more conducive to the circadian rhythm stability of critically ill patients.

## 6 Conclusion

Through this preliminary exploratory study, we observed that diversified nursing interventions were associated with the improvement of melatonin rhythm in sedated patients in the intensive care unit, which suggested that regulating light, noise, nutrition and workflow may have positive potential to improve the circadian rhythm disorder of patients.

However, due to the above limitations of this study, especially the lack of statistical control over a variety of key confounding factors and the source of potential bias, we cannot establish the causal relationship between intervention measures and effects. The condition of patients in intensive care unit is extremely complex, and the circadian rhythm is affected by multiple factors. Therefore, from the perspective of circadian rhythm to improve the patient’s condition, the obstruction of the channel is long.

Future research needs to adopt more rigorous design, such as large sample, multi center randomized controlled trials, and use multivariate statistical model to verify the specific efficacy and independent contribution of diversified nursing intervention on the basis of controlling the disease severity, drug use, delirium and other key confounding factors, so as to provide a more reliable basis for evidence-based nursing practice in ICU.

## Data Availability

The original contributions presented in the study are included in the article/supplementary material, further inquiries can be directed to the corresponding author/s.
